# The Aging Well Through Interaction and Scientific Education Program: An Adaptable Model for Brain Health Education

**DOI:** 10.1093/geroni/igaf122.1117

**Published:** 2025-12-31

**Authors:** Maureen O’Connor, Lauren Moo, Malissa Kraft, Jaye McLaren

**Affiliations:** Bedford Veterans Health Care System, Bedford, Massachusetts, United States; VA Bedford Healthcare System, Bedford, Massachusetts, United States; Bedford VA Medical Center, Bedford, Massachusetts, United States; VA Bedford Healthcare System, Bedford, Massachusetts, United States

## Abstract

The role of lifestyle factors such as diet, exercise, and socialization in brain health outcomes has been widely acknowledged. The contribution of attitudes about aging as they specifically relate to brain health outcomes and engagement in lifestyle activities has been less of a focus. The Aging Well through Interaction and Scientific Education (AgeWISE) program is a 12-week, manualized group that provides older adults with psychoeducation about brain aging, lifestyle factors associated with successful brain aging, and strategies to compensate for age-related cognitive change. A randomized controlled pilot study of forty-nine older (mean age=73), mostly Caucasian (98%), cognitively normal male Veterans revealed increases in memory contentment and sense of control over memory decline for AgeWISE participants. AgeWISE-Action Plan (AgeWISE-AP) is an expansion program developed to capitalize on and extend AgeWISE gains in memory controllability. To date, sixty-three older Veterans have been enrolled in AgeWISE-AP as part of an ongoing randomized controlled trial, with excellent (95%) retention in the intervention arm. AgeWISE has been adapted across settings (geriatric inpatient, synchronous telehealth), populations (non-VA, mild cognitive impairment), and cultures (non-US). We will describe AgeWISE and AgeWISE-AP, with a focus on the highly adaptable AgeWISE program, to illustrate how brain health programs can be implemented in clinical settings.

